# Maxillofacial trauma due to traffic accidents and falls: an exploratory study of associated factors

**DOI:** 10.4317/medoral.24229

**Published:** 2020-12-19

**Authors:** Damião Edgleys Porto, Yuri Wanderley Cavalcanti, Franklin Delano Soares Forte

**Affiliations:** 1 Post-Graduation in Dentistry, Health Sciences Center, Paraíba Federal University, campus universitário I, Brasil

## Abstract

**Background:**

This study aimed to determine the pattern of Maxillofacial trauma (MFT) due to traffic accidents and falls in a reference hospital in a rural region of northeastern Brazil between December 2011 and December 2018 and to identify associated factors.

**Material and Methods:**

This was a cross-sectional study using 585 medical records of patients with MFT. The data were subjected to a Poisson-Tweedie multiple regression analysis to estimate the Prevalence ratio (PR), with a 95% confidence interval (95% CI) and a significance level of *p*<0.05.

**Results:**

MFT due to traffic accidents was more prevalent among patients 21 to 40 years old (PR=2.30; 95% CI=1.20-4.41; *p*<0.001) diagnosed with zygomatic-orbital complex fractures (PR=1.80; 95% CI=1.08-2.98; *p*=0.023). Falls were more frequent among older groups of 41 to 60 years (PR=1.83; 95% CI=1.09-3.06; *p*=0.022) and over 61 years (PR=2.23; 95% CI=1.09-3.06; *p*=0.022). In traffic accidents, alcohol consumption increased the length of stay (PR=2.081; 95% CI=1.553-2.787; *p*<0.001), and patients who did not use personal protective equipment (PPE) had higher hospital costs (PR=179.964; 95% CI=1.485-1.994; *p*<0.001) for this etiology. Traffic accidents and falls are two of the main etiologies of MFT, especially for males in the young adult age group (traffic accidents) and those above 41 years (falls). Alcohol consumption and the nonuse of PPE influenced the length of the hospital stay and hospital costs.

**Conclusions:**

Strategies to confront this problem, such as road and highway improvements, effective enforcement of laws and intersectoral coordination involving the entire community to implement policies and prevention programs targeted at these populations, can be implemented.

** Key words:**Maxillofacial Injuries, public policy, hospital cost.

## Introduction

Maxillofacial trauma (MFT) is particularly important because it is responsible for a large fraction of emergency visits and the morbidity and mortality in trauma centers, which is reflected in a lack of hospital and polyclinic beds, in addition to the costs associated with the treatment, recovery and rehabilitation of these patients (1-4).

 Studies have shown that traffic accidents and falls are the most prevalent etiologies of MFT (5-9). These etiologies represent real challenges both for the management of public policies targeting the care of this population through the implementation of primary care programs focused on the prevention of the main etiological factors and the factors associated with MFT and for the care professionals involved in the treatment of increasingly severe injuries (1,5,6,7,9-11).

MFT related to traffic accidents is more common among males 21 to 40 years of age (1-3,5,8,9,11-19), while falls are the main causes of MFT in children and the elderly (5,10,11). Factors such as alcohol consumption (8,10,20), urban violence and the violation of traffic rules contribute to the increasing prevalence of MFT (8,12,13,14).

 The severity of MFT contributes to sequelae and severe and even fatal complications. In addition, MFT has social impacts: unemployment, low social support and more functional and esthetic problems due to numerous permanent defects, deformities and scars, which affects the individual’s quality of life and directly influences his or her return to social and productive life and reliance on social security programs (4,21-23). The discussion of this topic is relevant regarding the social security policies in Brazil and around the world.

Most studies (2,8,15,17) report the impact of MFT in large urban centers, demonstrating the need for studies in rural areas in northeastern Brazil, where surveillance and prevention of these injuries is more limited.

This study was focused on a regional hospital that is the main health unit of the Seridó and Curimataú region in the state of Paraíba, northeastern Brazil, and is a reference for the main medical-dentistry specialties. This hospital serves an average of 4,541 patients per month from 12 cities in the region.

Considering this scenario and the scarcity of epidemiological studies on these injuries in small cities in northeastern Brazil, the present study aimed to analyze the association between the patterns of MFT, length of stay, hospital costs and socioeconomic factors, alcohol consumption and use of PPE in this regional hospital in the Seridó and Curimataú region of the state of Paraíba, northeastern Brazil.

## Material and Methods

An analytical, quantitative, cross-sectional study was performed by analyzing the medical records of all patients with MFT in a regional public hospital of reference for emergency and trauma in a rural region of Northeast Brazil that performs approximately 133 urgent and elective surgeries per month. Of these, an average of 400 surgeries per year are due to MFT (http://sigtap.datasus.gov.br/tabela-unificada/app/sec/inicio.jsp).

The data were analyzed for the period from December 2011 to December 2018. The Oral and Maxillofacial Surgery and Traumatology service was established in December 2011. The Strengthening the Reporting of Observational Studies in Epidemiology Guidelines for observational studies were used to plan the study and report the results obtained.

All outpatient records and/or hospital records of patients with MFT were included, excluding those that were incomplete or struck through.

Data were collected using a form developed specifically for the study. The following independent variables were collected: sex, age range, skin color, occupation, family income, years of education, alcohol consumption, use of personal protective equipment (PPE) (seat belt, helmet), time of accident, and treatment. The following dependent variables were also collected: etiology of MFT, i.e., traffic accidents (motorcycle, automobile, and bicycle) and falls; length of hospital stay; and cost of the procedure (values obtained through the hospital administration from invoices from the Hospital Information System (http://www.saude.pb.gov.br/site/hospitais) during the studied period, considering hospitalization rates, medications and supplies, including osteosynthesis materials).

The variables “use of PPE”, “day of the week of the accident” and “time of accident” were only considered for MFT due to traffic accidents.

Pearson’s chi-square test was used to determine the bivariate associations between the dependent and the independent variables. For the analysis of the prevalence of MFT according to accidents and falls, the variables with significance at the 20% level (*p*<0.20) were included in the multivariate Poisson regression analysis with robust variance to estimate the crude and adjusted prevalence ratio (PR) with a confidence interval of 95%. Associations with *p*<0.05 were considered significant in all analyses. The goodness-of-fit of the final model was assessed using the Hosmer-Lemeshow test. Multivariate Tweedie regression analysis was used for the hospital costs and length of hospital stay, and the goodness-of-fit of the final model was tested by the Omnibus test. All the tests were performed in the Statistical Package for the Social Sciences (SPSS for Windows version 25.0; IBM Inc., Armonk, NY, USA).

## Results

From a total of 5,325 medical records, 604 (11.34%) included MFT, of which 19 were excluded because they contained incomplete data, resulting in 585 medical records. A total of 308 (52.6%) medical records included MFT due to traffic accidents, 173 (29.6%) were from falls and 104 (17.8%) were from physical aggression.

Table 1 describes the prevalence of MFT in traffic accidents. Most patients were male (68.5%), with a mean age of 33 years (standard deviation 8.1). There was a predominance of young adults in the of 21 to 40 year-old age group (50.3%), with an income of up to 1-2 minimum wage (49.7%) and more than eight years of formal education (62.0%).

**Table 1:**
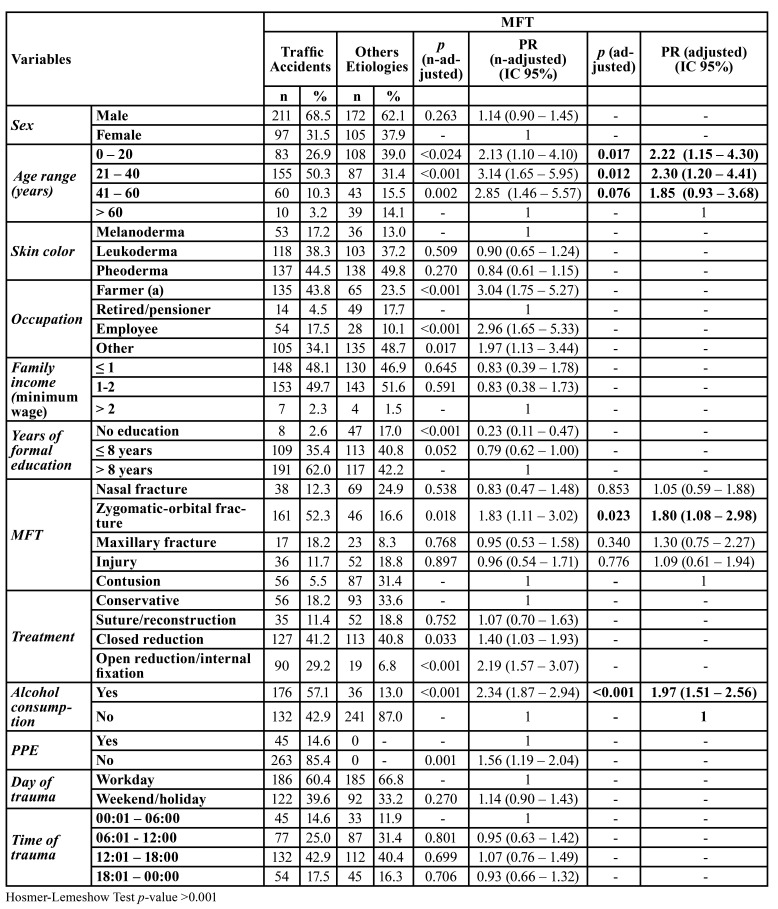
Multiple Poisson regression for the occurrence of MFT in traffic accidents, Brazil, 2019, n=585.

MFT occurred in all regions of the face in different proportions, and zygomatic-orbital complex fractures were the most prevalent in this study (52.3%). Closed reduction was the most commonly performed treatment (41.2%). Most patients had consumed alcohol (57.1%) and were not using PPE at the time of the accident (85.4%). MFT was most common on workdays (60.4%) between 12:01 and 18:00 (42.9%).

In the adjusted model, MFT due to traffic accidents was associated with patients 21 to 40 years old (PR=2.30; 95% CI=1.20-4.41; *p* <0.001) who had consumed alcohol (PR=1.97; 95% CI=1.51 - 2.56; *p* <0.001). Zygomatic-orbital complex fracture was the most prevalent (PR=1.80; 95% CI=1.08-2.98; *p* =0.023).

The prevalence of MFT due to falls is shown in Table 2. Most patients were male (56.6%) within 0 to 20 year-old age group (40.5%). Bruises (42.2%) and conservative treatment (45.7%) were prevalent. In the adjusted model, falls were associated with the age ranges of 41 to 60 years old (PR=1.83; 95% CI=1.09-3.06; *p* =0.022) and older than 61 years of age (PR=2.23, 95% CI 1.35-3.68), with no formal education (PR 2.08: 95% CI=1.31-3.29) or less than 8 years of formal education (PR=1.48: 95% CI=1.02-2.15), and with alcohol consumption (PR=0.21: 95% CI=0.12- 0.36).

**Table 2:**
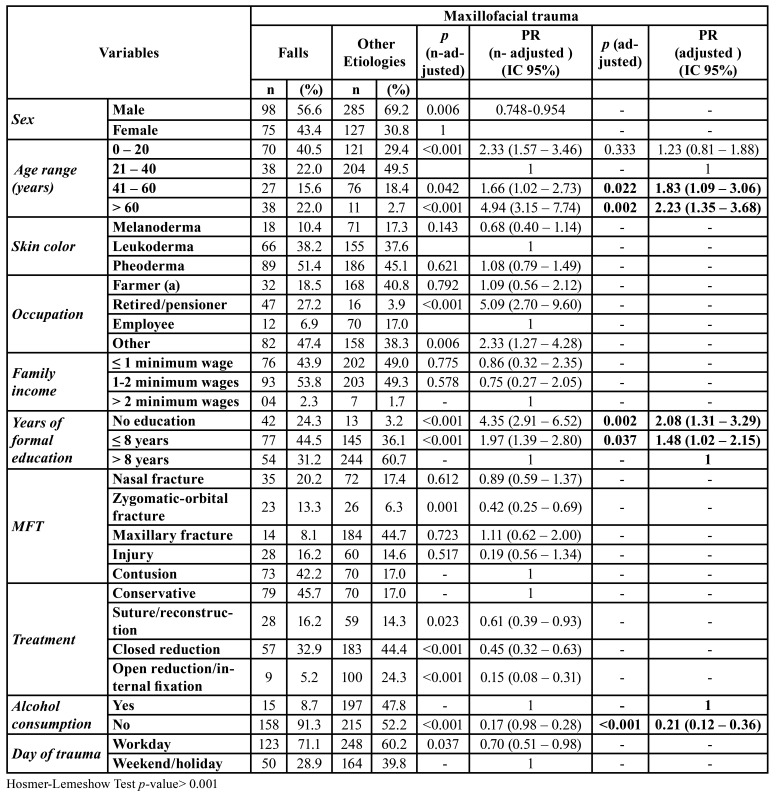
Multiple Poisson regression for the occurrence of Maxillofacial trauma in falls, Brazil, 2019, n=585.

A mean hospital stay of 1.18 days (standard deviation 1.65) was observed for MFT due to traffic accidents. In traffic accidents, patients who had consumed alcohol had longer hospital stays in the adjusted model (PR=2.081; 95% CI 1.553-2.787; *p* <0.001). In the case of falls, the age group influenced the length of the hospital stay, especially for patients that were older than 60 years of age (PR=0.542: 95% CI=0.296-0.992) and younger patients (0-20 years old) (PR=0.362: 95% CI 0.131 -0.999) (Table 3).

**Table 3:**
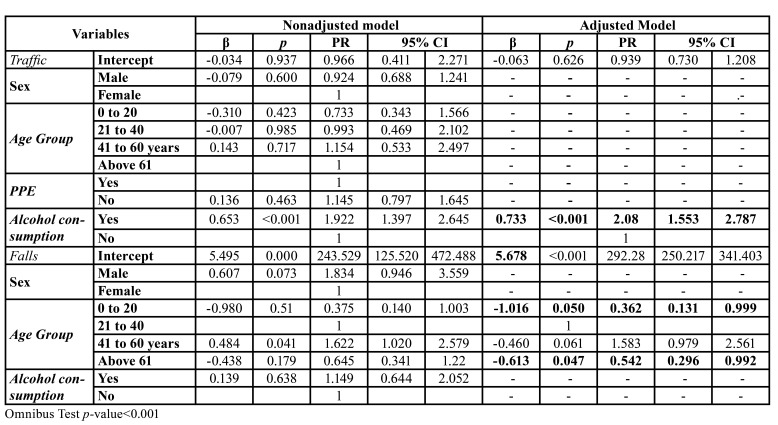
Multivariate Tweedie regression for the occurrence of Maxillofacial trauma and associated factors relative to length of hospital stay, Brazil, 2019.

The hospital costs of patients with MFT totaled USD 38,034.47, with an overall average cost of USD 65.02 per patient with MFT. For patients who were victims of traffic accidents, the total hospital cost was USD 29,888.60, with an average of USD 98.31 per patient. For patients who suffered falls, the hospital costs totaled USD 3,835.19, with an average of USD 22.17 per patient.

In the adjusted model, for traffic accidents, hospital costs were influenced by the nonuse of PPE (PR=179.964; 95% CI=1.485-1.994; *p*<0.001) (Table 4).

**Table 4:**
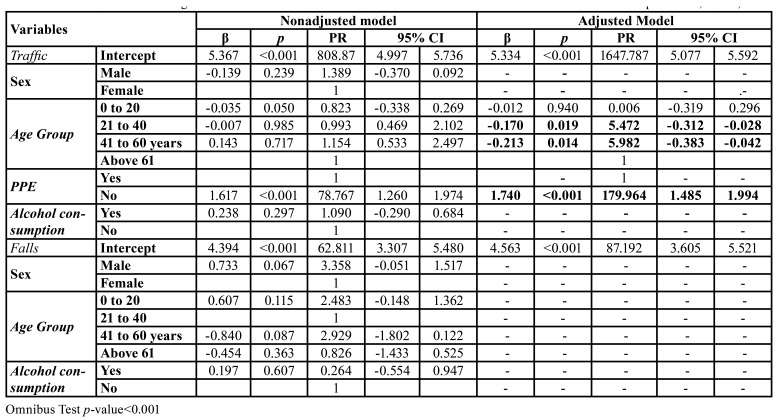
Multivariate Tweedie regression for the occurrence of Maxillofacial trauma and associated factors relative to hospital costs, Brazil, 2019.

## Discussion

This seven-year exploratory study showed that traffic accidents are the main etiological agent of MFT, followed by falls, similar to what has been reported in other studies (2,3,5-8,13-24). Traffic accidents and falls are two of the leading causes of trauma worldwide, including MFT (1,5,7,9-11).

In this study, most patients were male in the of 21 to 40 year-old age group. The literature indicates a greater tendency for MFT to be more prevalent in men in this age group (1-3,5,8,9,11-17,19), mainly because, in general, they more actively participate in social activities, sports activities, urban violence, traffic accidents, and drug use, including alcohol use.

Some authors (4,19,22) attribute the higher prevalence of traffic accidents to changes in habits and social dynamics that require the use of motor vehicles. In Brazil, these accidents are an important public health problem as a result of limited awareness of road safety, inadequate road conditions without improvement or expansion of the road network, the use of old vehicles without safety features, nonuse of seat belts or helmets, and consumption of alcohol or other drugs (2,8,15,19).

In the cities that compose the Seridó and Curimataú region of the state of Paraíba, which is the region of interest for this study, as well as in other small municipalities in Brazil, it is common for local management to prohibit the use of helmets in urban areas when there is no traffic surveillance agency, with the purpose of inhibiting the occurrence of robberies and enabling the identification of individuals. Moreover, in these municipalities, the consumption of alcoholic beverages and other drugs is very frequent. Leisure and urban spaces, which are essential for a better quality of life and health in a population, are lacking. Additionally, in these cities, the monitoring and punishment of the etiological factors and factors associated with MFT are more limited. Thus, the use of the existing social facilities are an opportunity for citizens to enhance their quality of life.

 Falls were the most prevalent cause of MFT in patients older than 41 years of age and especially those older than 61 years of age (1,5,9,11,23). The increased longevity of the population may lead to more severe repercussions on motor and sensory functions, such as the gait pattern, dynamic and semi-static postural control and balance, as well as a series of changes in physiological factors (e.g., obesity, decreased bone density, diabetes, and Alzheimer’s disease), coupled with the fact that patients in this age group have poorer reflexes (5,10,20,24).

Traffic accidents resulted in a higher prevalence of zygomatic-orbital complex fractures, corroborating the results of previous studies (6,7,9,13,18). Its prominent position on the face makes the zygomatic-orbital complex a common site for MFT, and its fracture is characterized by a solid body that transmits traumatic force to the sutures, which are fragile (25). In vehicles with additional safety devices, such as antilock braking systems (ABS) and air bags, this type of MFT may be present.

Bruises on the face were the most frequent MFT due to falls. In general, a low-impact etiology does not result in severe MFT in most cases (26).

For traffic accidents, surgical intervention, specifically closed reduction and open reduction, was the most commonly performed treatment. Regarding falls, conservative treatment was more often used. Studies (2,8,13,15,17-20) have shown that MFT due to traffic accidents is related to factors such as the type and severity of MFT, in addition to the degree of impact of the etiological agent. In general, traffic accidents cause high-impact injuries that result in MFT with more severe fractures that require surgical treatment. The opposite applies to falls in which the impact mechanism is smaller and causes less severe injuries, which often require conservative treatment.

Another factor related to the type of treatment used is the cost of the procedure (10,18). In general, conservative treatment does not require surgical intervention and, consequently, results in lower hospital costs, with no need for hospitalization. On the other hand, surgical treatment, mainly through open reduction, requires the use of an osteosynthesis material, which is a costly material, and combined with the longer hospital stays required for this type of treatment, this leads to a greater use of supplies and medications.

The mean length of the hospital stay was 1.18 days. The length of the hospital stay for traffic accidents was significantly influenced by the consumption of alcoholic beverages. This fact may be related to the neurophysiological effects of alcohol, which lead people to feel less fear and concern about the legal, physical or social consequences of their actions, in addition to impairing motor coordination and decision-making in drivers (20), resulting in increasingly severe trauma that requires more complex treatment and longer hospital stays (8).

The nonuse of PPE influenced the hospital costs related to traffic accidents. PPE (seat belt and helmet) help protect the craniofacial structures (6), so the nonuse of such equipment made the MFT more severe, resulting in greater surgical complexity and higher hospital costs.

Strategies and measures for controlling traffic accidents have been reported in Brazil. They include the Brazilian Traffic Code (abbreviated as CTB in Portuguese) established in 1998, and the Dry Law from 2008, which altered some parts of the CTB and implemented a zero blood-alcohol limit and harsher penalties for offenders (27). However, recklessness related to the consumption of alcoholic beverages and the disregard for the traffic laws by drivers still seems to persist, especially in the rural areas of the state of Paraíba and other neighboring states, where oversight is more limited and the Dry Law has not been effective.

It is important to strengthen the related legal provisions when seeking to reduce the occurrence of trauma, especially MFT, related to traffic accidents; therefore, collective intersectoral efforts are needed to enforce existing legal measures with permanent monitoring, including in small and rural municipalities (28,29).

This study has some limitations, such as its cross-sectional design, which does not allow the establishment of causal relationships. Blood alcohol tests were not conducted on patients to determine alcohol consumption, and both alcohol consumption and the use of PPE were self-reported. The study’s strengths include the fact that the data collection was standardized by the use of a single trained researcher, in addition to the periodic review of the database for errors.

The results of this study may support health (Ministry of Health, State and Municipal Health and Hospital Departments), traffic (Detran [State Traffic Department] and Ciretran [Brazil Regional District of Traffic]) and public safety (civil police, military and federal highway police) management agencies in developing public policies for prevention and better targeting of resources for this population. A specific example is the improvement of roads and highways, with better horizontal and vertical signage and more regular sidewalks, cycle paths and areas for pedestrians.

Data from this study may contribute to the improvement of the effectiveness of the legal measures instituted in small cities, the acquisition of supplies and equipment for the optimization of the diagnosis and treatment of MFT, and expansion and training of the professional teams to provide greater support for reference hospitals that provide care for these injuries in these municipalities.

## Conclusions

Traffic accidents and falls are important etiologies of MFT and are especially prevalent in males, predominantly affecting young adults in the case of traffic accidents and individuals over 41 years of age in the case of falls. Alcohol consumption and the nonuse of PPE influence the length of the hospital stay and the hospital costs. These results may be useful to local authorities and the entire population for developing strategies to address this problem, such as improvements in roads and highways, effective enforcement of laws, and intersectoral coordination involving the whole community for the implementation of prevention policies and strategies targeting this population.
